# Supplementary material for: Metabolomics Combined with Multivariate Statistical Analysis for Screening of Chemical Markers between Gentiana scabra and Gentiana rigescens
Source: Molecules. 2020 Mar 9;25(5):1228. doi: 10.3390/molecules25051228 (PMC7179410; doi:10.3390/molecules25051228)
Supplement: Supplementary file 1 [file molecules-25-01228-s001.pdf]

Metabolomics combined with multivariate statistical analysis for screening of chemical markers between *Gentiana scabra* and *Gentiana rigescens*

Gaole Zhang [a,b†], Yun Li [b,c†], Wenlong Wei [b], Jiayuan Li [b] and Haoju Li [b]

Yong Huang [b] and Dean Guo [a,b*]

Affiliation

Shanghai Research Center for Modernization of Traditional Chinese Medicine, National Engineering Laboratory for TCM Standardization Technology, Shanghai Institute of Materia Medica, Chinese Academy of Science, Shanghai, 201203, China

† These two authors contributed equally to this work

Correspondence:

Prof. De-an Guo, Shanghai Research Center for Modernization of Traditional Chinese Medicine, National Engineering Laboratory for TCM Standardization Technology, Shanghai Institute of Materia Medica, Chinese Academy of Sciences, Haike Road 501, Shanghai 201203, China.

Email: daguo@simm.ac.cn Phone: +86 21 50271516 Fax: +86 21 50272789

Table S1. The columns screened for separation of GRR.

Table S1. The columns screened for separation of GRR.

No.	Type	Specification	Separation mechanism	PH
1	Eclipse Plus C18	2.1×100 mm,1.8 μm	be used for a wide range of applications and over a pH range of 2–9	2-9
2	Zorbax Extend C18	2.1×100 mm,1.8 μm	high efficiency in applications with high pH values	2-11.5
3	BEH-C18	2.1×100 mm,1.8 μm	Suitable for the separation of complex compounds with high PH rang	1-12
4	HSS T3	2.1×100 mm,1.8 μm	Suitable for the separation of complex compounds with wide polar range	2-8
5	Kinetex XB C18	3.0×100 mm,1.7 μm	Good retention of polar compounds	1.5-10
6	Zobax SB C18	2.1×150 mm,1.8 μm	Resistant to high temperature,low pH values	1-8
